# 1-Phenyl­piperazine-1,4-diium tetra­chlorido­cobalt(II)

**DOI:** 10.1107/S1600536814005790

**Published:** 2014-03-22

**Authors:** Abdelhamid Chiheb Dhieb, Daron E. Janzen, Mohamed Rzaigui, Wajda Smirani Sta

**Affiliations:** a Laboratoire de Chimie des Matériaux, Faculté des Sciences de Bizerte, 7021 Zarzouna Bizerte, Tunisia; b Department of Chemistry and Biochemistry, St Catherine University, 2004 Randolph Avenue, #4282, St Paul, MN 55105, USA

## Abstract

In the title mol­ecular salt, (C_10_H_16_N_2_)[CoCl_4_], the piperazine ring of the phenyl­piperazine dication adopts a chair conformation and the phenyl ring occupies an equatorial orientation. In the tetra­chlorido­cobaltate(II) dianion, the Co—Cl bond lengths for the chloride ions not accepting hydrogen bonds are significantly shorter than those for the chloride ions accepting such bonds. In the crystal, the components are linked by N—H⋯Cl hydrogen bonds, generating [001] chains.

## Related literature   

For background to organic-inorganic hybrid materials, see: Bringley & Rajeswaran (2006 ▶); Brammer *et al.* (2002) ▶. For phenyl­piperazinium cations, see: Ben Garbia *et al.* (2005 ▶). For related structures, see: Mghandef & Boughzala (2014 ▶); Wang *et al.* (2012 ▶).
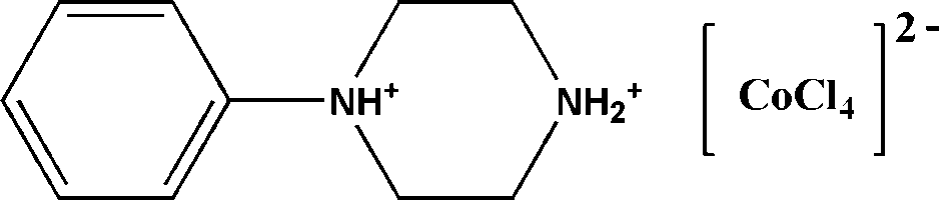


## Experimental   

### 

#### Crystal data  

(C_10_H_16_N_2_)[CoCl_4_]
*M* *_r_* = 365.00
Monoclinic, 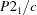

*a* = 7.7400 (9) Å
*b* = 20.278 (3) Å
*c* = 9.6257 (12) Å
β = 105.121 (8)°
*V* = 1458.5 (3) Å^3^
*Z* = 4
Mo *K*α radiation
μ = 1.89 mm^−1^
*T* = 173 K
0.46 × 0.32 × 0.28 mm

#### Data collection  

Rigaku XtaLAB mini diffractometer
Absorption correction: multi-scan (*REQAB*; Rigaku, 1998 ▶) *T*_min_ = 0.465, *T*_max_ = 0.589
15030 measured reflections
3335 independent reflections
3034 reflections with *F* ^2^ > 2σ(*F* ^2^)
*R*_int_ = 0.023

#### Refinement  

*R*[*F* ^2^ > 2σ(*F* ^2^)] = 0.023
*wR*(*F* ^2^) = 0.054
*S* = 1.14
3335 reflections
166 parameters
H atoms treated by a mixture of independent and constrained refinement
Δρ_max_ = 0.26 e Å^−3^
Δρ_min_ = −0.36 e Å^−3^

### 

Data collection: *CrystalClear-SM Expert* (Rigaku, 2011 ▶); cell refinement: *CrystalClear-SM Expert*; data reduction: *CrystalClear-SM Expert*; program(s) used to solve structure: *SIR2004* (Burla *et al.*, 2005 ▶); program(s) used to refine structure: *SHELXL97* (Sheldrick, 2008 ▶); molecular graphics: *ORTEP-3 for Windows* (Farrugia, 2012 ▶); software used to prepare material for publication: *CrystalStructure* (Rigaku, 2010 ▶).

## Supplementary Material

Crystal structure: contains datablock(s) General, I, 239R. DOI: 10.1107/S1600536814005790/hb7210sup1.cif

Structure factors: contains datablock(s) I. DOI: 10.1107/S1600536814005790/hb7210Isup2.hkl

CCDC references: 991791, 991792

Additional supporting information:  crystallographic information; 3D view; checkCIF report

## Figures and Tables

**Table 1:** Selected bond lengths (Å)

Co1—Cl1	2.3182 (5)
Co1—Cl2	2.2431 (5)
Co1—Cl3	2.2738 (5)
Co1—Cl4	2.2811 (6)

**Table 2:** Hydrogen-bond geometry (Å, °)

*D*—H⋯*A*	*D*—H	H⋯*A*	*D*⋯*A*	*D*—H⋯*A*
N1—H1*A*⋯Cl4	0.90 (3)	2.21 (3)	3.1043 (14)	173 (3)
N1—H1*B*⋯Cl1^i^	0.85 (2)	2.66 (3)	3.2963 (14)	132.2 (18)
N2—H2⋯Cl1^ii^	0.89 (3)	2.36 (2)	3.2262 (13)	164.6 (17)

Symmetry codes: (i) 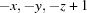; (ii) 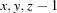.

**Fig. 1.**
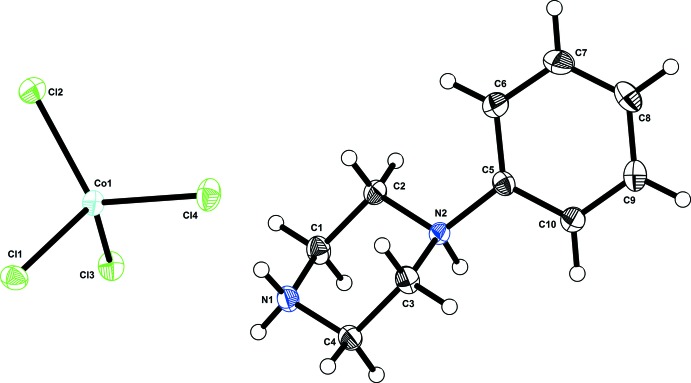
ORTEP-3 view of the title compound with displacement ellipsoids drawn at the 30% probability level.

**Fig. 2.**
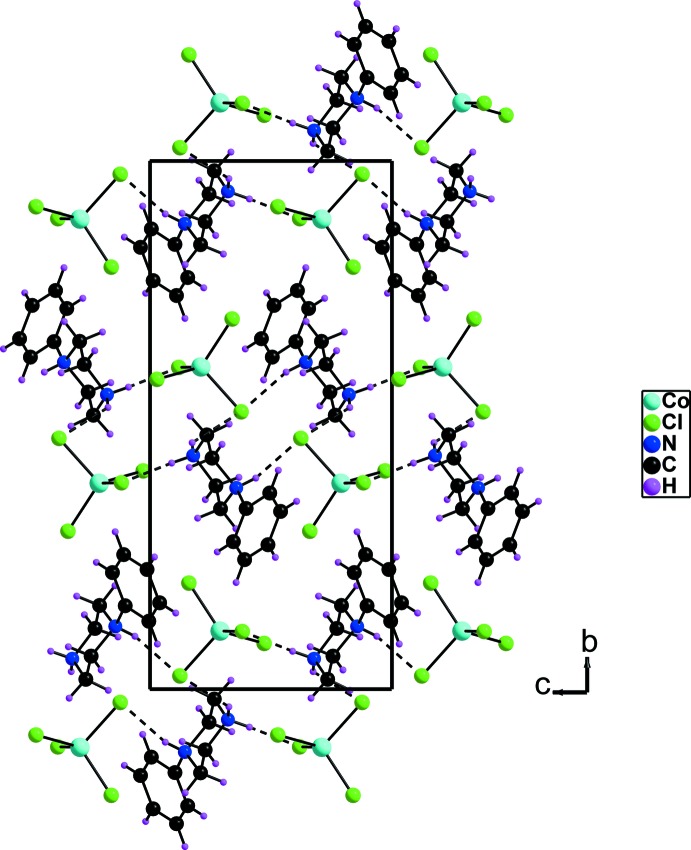
View of the atomic arrangement of the title compound along the a axis. Hydrogen bonds are shown as dashed lines.

**Fig. 3.**
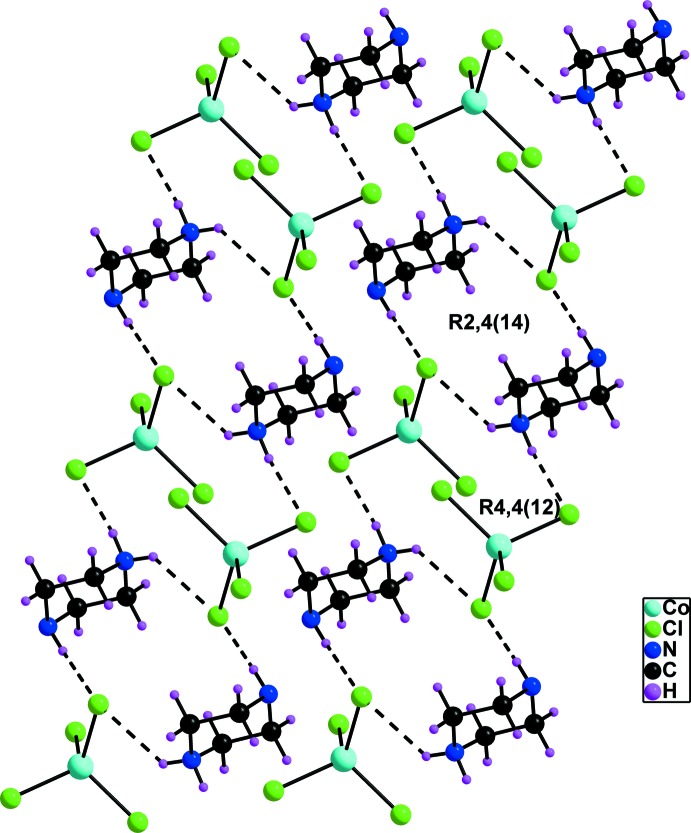
Graph-set description of ring types hydrogen bonding. Hydrogen bonds are shown as dashed lines.

## supplementary crystallographic information

### 1. Comment

Organic-inorganic hybrid materials have received extensive attention in recent years owing to their great fundamental and practical interest such as second-order nonlinear optical (NLO) responses, magnetism, luminescence, photography and drug delivery (Bringley & Rajeswaran, 2006). However, the energetics of NH····Cl—*M* (*M* = metal) hydrogen bonds and their possible roles in supramolecular chemistry have only been recently described in detail (Brammer *et al.*, 2002). It is therefore vital to design and synthesize new organic inorganic hybrid compound to explore their various properties.

The title inorganic-organic hybrid compound contains two basic components, the one [CoCl_4_]^2–^ anion and one (C_10_H_16_N_2_)^2+^ organic dication (Fig.1). The structure can be described by tetrachloridocobalt(II) units which form a hydrogen-bonded one-dimensional network with the phenylpiperazinium (N–H···Cl: 3.1043 (14) Å, 3.2963 (14) Å, 3.2262 (13) Å of infinite ribbons located at *y* = 0 and *y* = 1/2 that translate along the c direction (Fig. 2). The vander Waals contacts between these ribbons give rise to a three-dimensional network in the structure and add stability to this structure. In the [CoCl_4_] tetrahedra, the Co—Cl bond lengths and Cl–Co–Cl angles, ranging from 2.2431 (5) to 2.3182 (5) Å and from 103.983 (1) to 116.304 (19)° respectively, are in agreement with those found in 1-(4-hydroxyphenyl)piperazine-1,4-diium tetrachloridocobalt(II) monohydrate (Mghandef & Boughzala, 2014). The nearest Co···Co intra-chain distance is 6.044 Å, while that between adjacent chains is 7.441 Å. The organic cation, (C_10_H_16_N_2_)^2+^, contains a piperazindium ring in a chair conformation and a planar aromatic ring (atoms C5—C10 r.m.s. deviation = 0.003 Å). In this dication, the bond lengths of C5—C6, C5—C10, C6—C7, C7—C8, C8—C9 and C9—C10 are between single bonds and double bonds and are similar to those of benzene (Ben Garbia *et al.*, 2005). Furthermore, the distances N1—C4, N1—C1, N2—C5, N2—C2, N2—C3, C1—C2 and C3—C4 indicate single bonds.

In this compound, H1A and H2A attached to the N1 nitrogen atom and H2 attached to N2 nitrogen atom play an important role in forming the molecular association through hydrogen bonding. Here two chlorine atoms, Cl1 and Cl4, act as acceptors of N–H···Cl H-bonds. The chlorine atoms Cl2 and Cl3 do not participate in the hydrogen bonding network. The deviation from the perfect tetrahedral arrangement around Co(II) in the title compound can be explained by involvment of the chlorine ions in hydrogen bonding. Three different [CoCl_4_]^2–^ anions are act as hydrogen-bonding acceptors to each phenylpiperazinium dication forming two different hydrogen-bonding ring motifs, *R*_2_^4^(14) and *R*_4_^4^(12) (Fig.3). As Cl2 and Cl3 do not act as hydrogen-bond acceptors, the bond angles Cl2–Co–Cl3 and Cl2–Co–Cl4 (116.304 (19)° and 112.971 (19)°, respectively) display comparatively large deviations from the expected tetrahedral arrangement around Co(II). Similar features have been also observed in the structure of dimorpholinium tetrachloridocobaltate(II) (Wang *et al.*, 2012), where three chlorine atoms are engaged in the hydrogen-bonding network and distortions from tetrahedral predictions are present in the Cl–Co–Cl angles. The structure of dimorpholinium tetrachloridocobaltate(II) also displays a *R*_4_^4^(12) hydrogen bonding motif, but does not possess a second unique *R*_2_^4^(14) motif that the title compound does exhibit.

### 2. Experimental

A mixture of CoCl_2_·H_2_O and phenylpiperazine was dissolved in an aqueous solution of hydrochloric acid (molar ratio 1:1:1). The obtained solution was stirred for 2 h and then kept at room temperature. Blue prisms of the title compound were obtained two weeks later.

### 3. Refinement

Many hydrogen atoms were treated in calculated positions and refined in the model as riding with distances of C—H = 0.95 and 0.99 Å for the phenyl and methylene groups, respectively, and with *U*_iso_(H) = k×*U*_eq_(C), k = 1.2. Hydrogen atoms H1A, H1B, and H2 were located in the electron density map, and their positions were refined with *U*_iso_(H) = k×*U*_eq_(C), k =1.2.

### Crystal data

**Table:**

(C_10_H_16_N_2_)[CoCl_4_]	*F*(000) = 740.00
*M**_r_* = 365.00	*D*_x_ = 1.662 Mg m^−^^3^
Monoclinic, *P*2_1_/*c*	Mo *K*α radiation, λ = 0.71075 Å
Hall symbol: -P 2ybc	Cell parameters from 14000 reflections
*a* = 7.7400 (9) Å	θ = 3.0–27.6°
*b* = 20.278 (3) Å	µ = 1.89 mm^−^^1^
*c* = 9.6257 (12) Å	*T* = 173 K
β = 105.121 (8)°	Prism, blue
*V* = 1458.5 (3) Å^3^	0.46 × 0.32 × 0.28 mm
*Z* = 4	

### Data collection

**Table:**

Rigaku XtaLAB mini diffractometer	3034 reflections with *F*^2^ > 2σ(*F*^2^)
Detector resolution: 6.849 pixels mm^-1^	*R*_int_ = 0.023
ω scans	θ_max_ = 27.5°
Absorption correction: multi-scan (*REQAB*; Rigaku, 1998)	*h* = −10→10
*T*_min_ = 0.465, *T*_max_ = 0.589	*k* = −26→26
15030 measured reflections	*l* = −12→12
3335 independent reflections	

### Refinement

**Table:**

Refinement on *F*^2^	Secondary atom site location: difference Fourier map
*R*[*F*^2^ > 2σ(*F*^2^)] = 0.023	Hydrogen site location: inferred from neighbouring sites
*wR*(*F*^2^) = 0.054	H atoms treated by a mixture of independent and constrained refinement
*S* = 1.14	*w* = 1/[σ^2^(*F*_o_^2^) + (0.0197*P*)^2^ + 0.6924*P*] where *P* = (*F*_o_^2^ + 2*F*_c_^2^)/3
3335 reflections	(Δ/σ)_max_ = 0.001
166 parameters	Δρ_max_ = 0.26 e Å^−^^3^
0 restraints	Δρ_min_ = −0.36 e Å^−^^3^
Primary atom site location: structure-invariant direct methods	

### Special details

**Table:**

Geometry. ENTER SPECIAL DETAILS OF THE MOLECULAR GEOMETRY
Refinement. Refinement was performed using all reflections. The weighted *R*-factor (*wR*) and goodness of fit (*S*) are based on *F*^2^. *R*-factor (gt) are based on *F*. The threshold expression of *F*^2^ > 2.0 σ(*F*^2^) is used only for calculating *R*-factor (gt).

### Fractional atomic coordinates and isotropic or equivalent isotropic displacement parameters (Å^2^)

**Table:**

	*x*	*y*	*z*	*U*_iso_*/*U*_eq_	
Co1	0.14256 (3)	0.110059 (11)	0.71031 (2)	0.01829 (7)	
Cl1	0.17830 (5)	0.025120 (19)	0.87659 (4)	0.02225 (9)	
Cl2	0.12699 (6)	0.20091 (2)	0.83944 (4)	0.02466 (10)	
Cl3	−0.10305 (5)	0.08669 (2)	0.52831 (4)	0.02264 (9)	
Cl4	0.38614 (5)	0.10918 (2)	0.61855 (4)	0.02585 (10)	
N1	0.16483 (19)	0.05757 (7)	0.32178 (15)	0.0196 (3)	
N2	0.36753 (17)	0.11889 (6)	0.14374 (14)	0.0156 (3)	
C1	0.0927 (3)	0.11496 (8)	0.22774 (18)	0.0212 (4)	
C2	0.2443 (2)	0.15844 (8)	0.20870 (18)	0.0195 (4)	
C3	0.4446 (2)	0.06256 (8)	0.24379 (18)	0.0207 (4)	
C4	0.2953 (3)	0.01865 (8)	0.26558 (18)	0.0213 (4)	
C5	0.5097 (2)	0.15791 (8)	0.10288 (16)	0.0171 (3)	
C6	0.5541 (3)	0.22071 (9)	0.15569 (19)	0.0255 (4)	
C7	0.6844 (3)	0.25474 (9)	0.1091 (2)	0.0291 (4)	
C8	0.7669 (3)	0.22656 (9)	0.01261 (19)	0.0261 (4)	
C9	0.7195 (3)	0.16350 (9)	−0.03960 (18)	0.0234 (4)	
C10	0.5899 (3)	0.12865 (8)	0.00517 (18)	0.0204 (4)	
H1C	0.0121	0.1409	0.2714	0.0255*	
H1D	0.0222	0.0990	0.1326	0.0255*	
H1A	0.221 (3)	0.0719 (12)	0.411 (3)	0.040 (7)*	
H1B	0.076 (3)	0.0334 (11)	0.326 (3)	0.027 (6)*	
H2A	0.1951	0.1961	0.1451	0.0234*	
H2B	0.3115	0.1762	0.3032	0.0234*	
H2	0.305 (3)	0.1003 (10)	0.063 (3)	0.025 (5)*	
H3A	0.5136	0.0803	0.3377	0.0248*	
H3B	0.5271	0.0364	0.2024	0.0248*	
H4A	0.2329	−0.0020	0.1729	0.0256*	
H4B	0.3465	−0.0169	0.3344	0.0256*	
H6	0.4972	0.2401	0.2220	0.0306*	
H7	0.7172	0.2980	0.1441	0.0349*	
H8	0.8561	0.2503	−0.0181	0.0313*	
H9	0.7761	0.1442	−0.1062	0.0281*	
H10	0.5565	0.0855	−0.0303	0.0245*	

### Atomic displacement parameters (Å^2^)

**Table:**

	*U*^11^	*U*^22^	*U*^33^	*U*^12^	*U*^13^	*U*^23^
Co1	0.01922 (12)	0.02023 (12)	0.01622 (11)	0.00007 (8)	0.00605 (9)	0.00011 (8)
Cl1	0.0271 (2)	0.01947 (19)	0.01833 (18)	−0.00352 (15)	0.00258 (15)	0.00167 (15)
Cl2	0.0297 (3)	0.0210 (2)	0.0247 (2)	0.00116 (16)	0.00976 (17)	−0.00198 (16)
Cl3	0.01909 (19)	0.0276 (2)	0.02077 (19)	−0.00029 (15)	0.00430 (15)	0.00099 (16)
Cl4	0.0190 (2)	0.0383 (3)	0.0214 (2)	−0.00280 (16)	0.00730 (16)	−0.00424 (17)
N1	0.0193 (7)	0.0222 (8)	0.0189 (7)	−0.0024 (6)	0.0078 (6)	0.0004 (6)
N2	0.0167 (7)	0.0167 (7)	0.0139 (7)	−0.0018 (5)	0.0048 (5)	−0.0016 (5)
C1	0.0188 (8)	0.0235 (9)	0.0228 (9)	0.0029 (7)	0.0079 (7)	0.0035 (7)
C2	0.0208 (8)	0.0184 (8)	0.0217 (8)	0.0023 (6)	0.0095 (7)	−0.0008 (7)
C3	0.0192 (8)	0.0211 (9)	0.0232 (8)	0.0038 (7)	0.0080 (7)	0.0050 (7)
C4	0.0243 (9)	0.0180 (8)	0.0243 (8)	0.0009 (7)	0.0111 (7)	0.0019 (7)
C5	0.0160 (8)	0.0194 (8)	0.0155 (8)	−0.0018 (6)	0.0033 (6)	0.0018 (6)
C6	0.0285 (9)	0.0239 (9)	0.0267 (9)	−0.0053 (7)	0.0118 (8)	−0.0064 (8)
C7	0.0293 (10)	0.0231 (9)	0.0360 (10)	−0.0087 (7)	0.0106 (8)	−0.0050 (8)
C8	0.0204 (9)	0.0296 (10)	0.0285 (9)	−0.0056 (7)	0.0066 (7)	0.0064 (8)
C9	0.0208 (8)	0.0307 (10)	0.0204 (8)	0.0016 (7)	0.0082 (7)	0.0019 (7)
C10	0.0209 (8)	0.0208 (8)	0.0201 (8)	−0.0007 (7)	0.0062 (7)	−0.0011 (7)

### Geometric parameters (Å, º)

**Table:**

Co1—Cl1	2.3182 (5)	N1—H1A	0.90 (3)
Co1—Cl2	2.2431 (5)	N1—H1B	0.86 (3)
Co1—Cl3	2.2738 (5)	N2—H2	0.89 (2)
Co1—Cl4	2.2811 (6)	C1—H1C	0.990
N1—C1	1.491 (2)	C1—H1D	0.990
N1—C4	1.490 (3)	C2—H2A	0.990
N2—C2	1.502 (3)	C2—H2B	0.990
N2—C3	1.513 (2)	C3—H3A	0.990
N2—C5	1.489 (3)	C3—H3B	0.990
C1—C2	1.517 (3)	C4—H4A	0.990
C3—C4	1.516 (3)	C4—H4B	0.990
C5—C6	1.381 (3)	C6—H6	0.950
C5—C10	1.388 (3)	C7—H7	0.950
C6—C7	1.389 (3)	C8—H8	0.950
C7—C8	1.381 (3)	C9—H9	0.950
C8—C9	1.388 (3)	C10—H10	0.950
C9—C10	1.385 (3)		
			
Cl1—Co1—Cl2	103.983 (19)	N1—C1—H1C	109.580
Cl1—Co1—Cl3	107.597 (18)	N1—C1—H1D	109.572
Cl1—Co1—Cl4	107.440 (18)	C2—C1—H1C	109.578
Cl2—Co1—Cl3	116.304 (19)	C2—C1—H1D	109.582
Cl2—Co1—Cl4	112.971 (19)	H1C—C1—H1D	108.117
Cl3—Co1—Cl4	108.021 (19)	N2—C2—H2A	109.754
C1—N1—C4	112.03 (14)	N2—C2—H2B	109.749
C2—N2—C3	109.03 (13)	C1—C2—H2A	109.748
C2—N2—C5	114.81 (12)	C1—C2—H2B	109.747
C3—N2—C5	111.93 (12)	H2A—C2—H2B	108.227
N1—C1—C2	110.37 (13)	N2—C3—H3A	109.634
N2—C2—C1	109.60 (13)	N2—C3—H3B	109.651
N2—C3—C4	110.07 (12)	C4—C3—H3A	109.646
N1—C4—C3	110.63 (14)	C4—C3—H3B	109.646
N2—C5—C6	121.53 (16)	H3A—C3—H3B	108.161
N2—C5—C10	116.34 (14)	N1—C4—H4A	109.525
C6—C5—C10	122.09 (17)	N1—C4—H4B	109.519
C5—C6—C7	118.12 (18)	C3—C4—H4A	109.521
C6—C7—C8	120.89 (17)	C3—C4—H4B	109.518
C7—C8—C9	120.01 (18)	H4A—C4—H4B	108.083
C8—C9—C10	120.14 (18)	C5—C6—H6	120.943
C5—C10—C9	118.76 (16)	C7—C6—H6	120.939
C1—N1—H1A	109.7 (15)	C6—C7—H7	119.555
C1—N1—H1B	107.3 (13)	C8—C7—H7	119.550
C4—N1—H1A	108.0 (16)	C7—C8—H8	119.998
C4—N1—H1B	110.1 (15)	C9—C8—H8	119.996
H1A—N1—H1B	110 (3)	C8—C9—H9	119.930
C2—N2—H2	109.3 (15)	C10—C9—H9	119.935
C3—N2—H2	105.8 (13)	C5—C10—H10	120.626
C5—N2—H2	105.5 (15)	C9—C10—H10	120.617
			
C1—N1—C4—C3	55.10 (15)	N1—C1—C2—N2	58.69 (16)
C4—N1—C1—C2	−56.04 (16)	N2—C3—C4—N1	−56.76 (16)
C2—N2—C3—C4	59.86 (14)	N2—C5—C6—C7	−178.07 (11)
C3—N2—C2—C1	−60.67 (13)	N2—C5—C10—C9	178.25 (11)
C2—N2—C5—C6	16.73 (17)	C6—C5—C10—C9	0.5 (3)
C2—N2—C5—C10	−161.00 (11)	C10—C5—C6—C7	−0.5 (3)
C5—N2—C2—C1	172.84 (10)	C5—C6—C7—C8	0.1 (3)
C3—N2—C5—C6	−108.25 (14)	C6—C7—C8—C9	0.2 (3)
C3—N2—C5—C10	74.02 (15)	C7—C8—C9—C10	−0.2 (3)
C5—N2—C3—C4	−172.01 (11)	C8—C9—C10—C5	−0.2 (3)

### Hydrogen-bond geometry (Å, º)

**Table:**

*D*—H···*A*	*D*—H	H···*A*	*D*···*A*	*D*—H···*A*
N1—H1*A*···Cl4	0.90 (3)	2.21 (3)	3.1043 (14)	173 (3)
N1—H1*B*···Cl1^i^	0.85 (2)	2.66 (3)	3.2963 (14)	132.2 (18)
N2—H2···Cl1^ii^	0.89 (3)	2.36 (2)	3.2262 (13)	164.6 (17)

Symmetry codes: (i) −*x*, −*y*, −*z*+1; (ii) *x*, *y*, *z*−1.

## References

[bb1] Ben Garbia, I., Kefi, R., Rayes, A. & Ben Nasr, C. (2005). *Z. Kristallogr. New Cryst. Struct.* **220**, 333–334.

[bb11] Brammer, L., Swearingen, J. K., Bruton, E. A. & Sherwood, P. (2002). *Proc Natl Acad Sci USA*, **99**, 4956–4961. 10.1073/pnas.072623399 PMC122702 11959946

[bb2] Bringley, J. F. & Rajeswaran, M. (2006). *Acta Cryst.* E**62**, m1304–m1305.

[bb3] Burla, M. C., Caliandro, R., Camalli, M., Carrozzini, B., Cascarano, G. L., De Caro, L., Giacovazzo, C., Polidori, G. & Spagna, R. (2005). *J. Appl. Cryst.* **38**, 381–388.

[bb4] Farrugia, L. J. (2012). *J. Appl. Cryst.* **45**, 849–854.

[bb5] Mghandef, M. & Boughzala, H. (2014). *Acta Cryst.* E**70**, m75. 10.1107/S1600536814001767 PMC3998272 24764833

[bb6] Rigaku (1998). *REQAB* Rigaku Corporation, Tokyo, Japan.

[bb7] Rigaku (2010). *CrystalStructure* Rigaku Corporation, Tokyo, Japan.

[bb8] Rigaku (2011). *CrystalClear-SM Expert* Rigaku Corporation, Tokyo, Japan.

[bb9] Sheldrick, G. M. (2008). *Acta Cryst.* A**64**, 112–122. 10.1107/S0108767307043930 18156677

[bb10] Wang, W.-Z., Ismayilov, R. H., Lee, G.-H., Wen, Y.-S. & Peng, S.-M. (2012). *Acta Cryst.* E**68**, m51. 10.1107/S1600536811053128 PMC3254319 22259351

